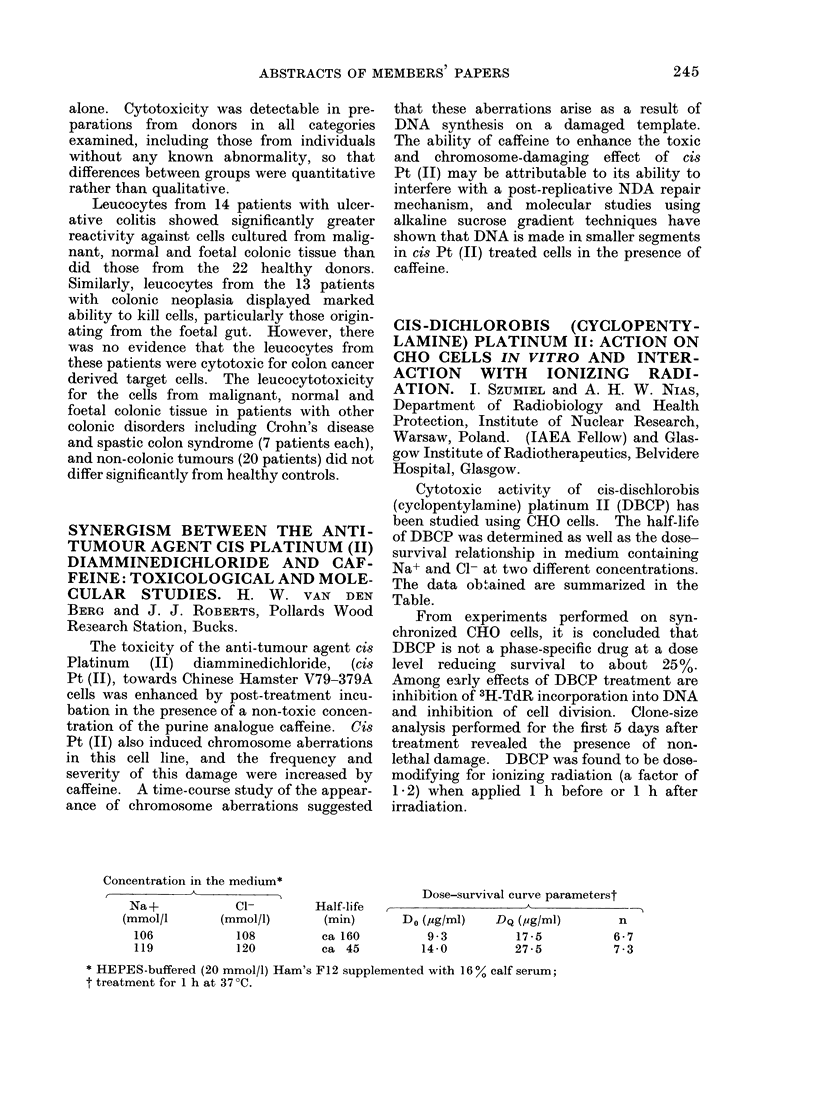# Proceedings: Cis-dichlorobis (cyclopentylamine) platinum II: action on CHO cells in vitro and interaction with ionizing radiation.

**DOI:** 10.1038/bjc.1975.172

**Published:** 1975-08

**Authors:** I. Szumiel, A. H. Nias


**CIS-DICHLOROBIS (CYCLOPENTYLAMINE) PLATINUM II: ACTION ON CHO CELLS *IN VITRO* AND INTERACTION WITH IONIZING RADIATION.** I. Szumiel and A. H. W. Nias, Department of Radiobiology and Health Protection, Institute of Nuclear Research, Warsaw, Poland. (IAEA Fellow) and Glasgow Institute of Radiotherapeutics, Belvidere Hospital, Glasgow.

Cytotoxic activity of cis-dischlorobis (cyclopentylamine) platinum II (DBCP) has been studied using CHO cells. The half-life of DBCP was determined as well as the dose–survival relationship in medium containing $Na^+$ and $Cl^-$ at two different concentrations. The data obtained are summarized in the Table.

From experiments performed on synchronized CHO cells, it is concluded that DBCP is not a phase-specific drug at a dose level reducing survival to about 25%. Among early effects of DBCP treatment are inhibition of $^3$H-TdR incorporation into DNA and inhibition of cell division. Clone-size analysis performed for the first 5 days after treatment revealed the presence of non-lethal damage. DBCP was found to be dose-modifying for ionizing radiation (a factor of 1·2) when applied 1 h before or 1 h after irradiation.

| Concentration in the medium* | | | Dose–survival curve parameters† | | |
|---|---|---|---|---|---|
| Na+ (mmol/l | $Cl^-$ (mmol/l) | Half-life (min) | $D_0$ (μg/ml) | $D_Q$ (μg/ml) | n |
| 106 | 108 | ca 160 | 9·3 | 17·5 | 6·7 |
| 119 | 120 | ca 45 | 14·0 | 27·5 | 7·3 |

* HEPES-buffered (20 mmol/l) Ham's F12 supplemented with 16% calf serum;
† treatment for 1 h at 37 °C.